# The LDL/HDL ratio predicts long-term risk of coronary revascularization in ST-segment elevation myocardial infarction patients undergoing percutaneous coronary intervention: a cohort study

**DOI:** 10.1590/1414-431X2021e11850

**Published:** 2022-02-04

**Authors:** Ruochen Zhang, Yan Fan, Yanbo Xue, Yunfei Feng, Caijuan Dong, Yamei Wang, Puqing Kou, Guoli Li, Aiqun Ma, Tingzhong Wang

**Affiliations:** 1 Department of Cardiovascular Medicine, the First Affiliated Hospital of Xi'an Jiaotong University, Xi'an Jiaotong University Medical College, Xi'an Jiaotong University, Xi'an, Shaanxi, China; 2 Key Laboratory of Environment and Genes Related to Diseases of Ministry of Education, Key Laboratory of Molecular Cardiology of Shaanxi Province, Institute of Cardiovascular Medicine of Xi'an Jiaotong University, Xi'an, Shaanxi, China; 3 Department of Cardiovascular Medicine, Gansu Provincial Hospital, Lanzhou, Gansu, China

**Keywords:** ST-segment elevation myocardial infarction, Percutaneous coronary intervention, Long-term prognosis, LDL/HDL ratio, Coronary revascularization

## Abstract

Clinical indicators do not adequately predict the long-term prognosis of patients with ST-segment elevation myocardial infarction (STEMI) following percutaneous coronary intervention (PCI). The low-density lipoprotein (LDL)/high-density lipoprotein (HDL) ratio is expected to be a reliable predictor of the long-term prognosis of these patients. This study aimed to explore the correlation between the LDL/HDL ratio and long-term prognosis in STEMI patients undergoing PCI. Patients with confirmed STEMI who underwent PCI in 7 hospitals in China from January 2009 to December 2011 were enrolled. Information about clinical endpoints, including all-cause death and major adverse cardiovascular events, was collected. Overall, 915 patients were included for analysis, the average follow-up time was 112.2 months. According to the LDL/HDL ratio, the patients were divided into 3 groups using the three-quantile method: low (LDL/HDL≤1.963), medium (1.963<LDL/HDL<2.595), and high (LDL/HDL≥2.595) LDL/HDL groups. The rate of coronary revascularization was higher in the high LDL/HDL group (28.52%) than in the low (17.38%, P=0.001) and medium (19.34%, P=0.010) LDL/HDL groups. The hazard ratio of coronary revascularization was significantly higher in the high LDL/HDL group than in the low (P=0.007) and medium (P=0.004) LDL/HDL groups. Increased LDL/HDL ratio was an independent risk factor for long-term coronary revascularization in STEMI patients undergoing PCI (HR=1.231, 95%CI: 1.023-1.482, P=0.028). These findings suggest that an increased LDL/HDL ratio was an independent risk factor for long-term coronary revascularization in STEMI patients undergoing PCI. The risk of coronary revascularization was significantly increased in patients with LDL/HDL≥2.595.

## Introduction

In low- and middle-income countries, mortality due to coronary heart disease will exceed that of infectious diseases by 2030, and coronary heart disease will become the disease with the highest mortality rate (1). ST-segment elevation myocardial infarction (STEMI) is one of the most serious types of coronary heart disease, and although percutaneous coronary intervention (PCI) can reduce the in-hospital mortality of STEMI patients, the long-term survival of STEMI patients has not improved (2). A cohort study conducted in Denmark showed that the 1-year all-cause death and cardiac death of STEMI patients who underwent PCI were 11.4 and 8.4%, respectively, and that the 5-year all-cause death and cardiac death were as high as 23.3 and 13.8%, respectively (3). Statistics from China also showed that the hospitalization rate of STEMI patients increased nearly four-fold from 2001 to 2011 (4). Therefore, exploring the risk factors that affect the long-term prognosis of STEMI patients after PCI and identifying early predictive indicators are of great interest.

At present, there is a lack of clinical indicators that can accurately predict the long-term prognosis of STEMI patients undergoing PCI. Commonly used methods for assessing the long-term prognosis of STEMI patients, such as coronary angiography and coronary computed tomography angiography (CTA), are mostly invasive and relatively expensive. On the other hand, biomarkers, such as serum low-density lipoprotein (LDL), high-density lipoprotein (HDL), glucose, and creatinine, are insufficient to accurately identify high-risk STEMI patients.

Many studies have shown that oxidation plays an important role in the occurrence and development of coronary heart disease (5). Not only are LDL and HDL important biomarkers of lipid metabolism, but the LDL/HDL ratio has also been confirmed to reflect the oxidation level (6). Therefore, the LDL/HDL ratio is expected to be a reliable indicator for predicting the long-term prognosis of STEMI patients. Previous studies have shown that the LDL/HDL ratio is an independent risk factor for cardiovascular adverse events in patients with acute coronary syndrome or acute myocardial infarction (7,8). However, research is still needed to explore the predictive value of the LDL/HDL ratio for the prognosis of STEMI patients.

In this research, a cohort study was used to explore the correlation between the LDL/HDL ratio and the long-term prognosis of STEMI patients who underwent PCI and to clarify the predictive value of the LDL/HDL ratio for the long-term prognosis of these patients. It is expected that the results of the study will provide new clinical indicators to accurately predict the prognosis of STEMI patients, thereby promoting early identification and early intervention for high-risk STEMI patients and improving patient prognosis.

## Material and Methods

### Study population

This was a multicenter observational study involving 7 hospitals in China. Patients aged >18 years with confirmed STEMI who underwent PCI between January 2009 and December 2011 were included. The diagnosis of STEMI followed the 2007 American College of Cardiology Foundation/American Heart Association guidelines (9). The diagnostic criteria were as follows: ≥1 persistent symptom of ischemia ≥30 min; ST-segment elevation ≥1 mm in ≥2 adjacent limb leads or ≥2 mm in ≥2 contiguous precordial leads, the presence of a new or suspicious new left bundle branch block, or the development of pathological Q waves on electrocardiography; and ≥1 episode of elevated serum biomarkers of myocardial necrosis (elevated creatine kinase and creatine kinase-myocardial band >2× the upper limit of normal, or elevated cardiac troponins). Patients were excluded if they had idiopathic cardiomyopathy, congenital heart disease, valvular heart disease, rheumatic or autoimmune disease, malignant tumors, severe liver or kidney dysfunction, or other uncontrollable systemic diseases; if their coronary anatomy was not amenable to PCI; or if they had an unsuccessful procedure. This study was approved by the Ethics Committee of the First Affiliated Hospital of Xi’an Jiaotong University, under approval number 2013-120, and informed consent was obtained from all participants. The study was registered in the Chinese Clinical Trial Registry (ChiCTR) (http://www.chictr.org) under identifier ChiCTR-PRCH-13003570. This study was performed in accordance with the guidelines of the Declaration of Helsinki.

### Baseline data collection

For all patients, demographic characteristics, medical history, laboratory measurements, echocardiography findings, angiographic information, and cardiovascular medications were obtained from the hospital medical record system. Blood samples and serum biomarkers such as LDL, HDL, total cholesterol (TC), triglycerides (TG), cystatin C (Cys C), and homocysteine (HCY) were collected within 1 hour of admission as a part of routine clinical care. All patients underwent color Doppler echocardiography within 72 h of admission.

### Follow-up

All patients had a face-to-face intensive midterm follow-up from August 2013 to January 2014 to obtain information about their clinical endpoints. In November 2019, the patients' clinical endpoints were counted by telephone and medical records.

### Clinical endpoint definition

The clinical endpoints included all-cause death and major adverse cardiovascular events (MACEs). MACEs included cardiac death, coronary revascularization, nonfatal acute myocardial infarction, heart failure, and stroke. Cardiac death was defined as death without a clear non-cardiac cause; coronary revascularization was the revascularization of any disease caused by ischemic symptoms or events, including PCI and coronary artery bypass grafting (CABG). The diagnosis of nonfatal acute myocardial infarction was based on the standards proposed by the Academic Research Consortium (ARC) (10). The diagnosis of heart failure was based on the diagnostic criteria recommended in the Chinese guidelines for the diagnosis and management of heart failure (11). Stroke referred to newly diagnosed cerebral infarction or hemorrhage.

### Statistical analysis

Categorical variables are reported as rates or percentages, and the chi-squared test was used for comparisons between groups. Continuous variables conforming to a normal distribution are reported as means±SD, and the independent-sample*t*-test was used for comparisons between groups. Continuous variables with skewed distributions are reported as medians (interquartile 1, interquartile 3), and the Mann-Whitney U test was used for comparisons between groups. The cumulative hazard curve was displayed by the Kaplan-Meier (K-M) method, and the log-rank test was used for comparisons between groups. The relationship between the LDL/HDL ratio and clinical endpoints was analyzed by Cox regression analysis. Pearson correlation analysis was used to determine the correlation between the LDL/HDL ratio and Cys C and HCY. A two-tailed P value of less than 0.05 was considered statistically significant. All statistical analyses were performed using SPSS 22.0 (SPSS, Inc., USA) and GraphPad Prism 7.0 (GraphPad Software, Inc., USA).

## Results

### Baseline data of the study population

After excluding 185 patients who were lost to follow-up, a total of 915 patients were included for analysis. Among them, 161 (17.60%) died, 440 (48.09%) had MACEs, and the average follow-up time was 112.2 months. According to the LDL/HDL ratio, 915 STEMI patients were divided into 3 groups using the three-quantile method: low LDL/HDL group (LDL/HDL≤1.963), medium LDL/HDL group (1.963<LDL/HDL<2.595), and high LDL/HDL group (LDL/HDL≥2.595).

The comparison of baseline data among the three groups is shown inTable 1. Compared with participants in the low LDL/HDL group, patients in the high LDL/HDL group were younger (P<0.001) and had a higher body mass index (BMI) (P<0.001), and the high LDL/HDL group had a higher proportion of patients with Killip grade I than the low LDL/HDL group (P=0.005). In terms of laboratory tests, blood glucose (P=0.008) and TG, TC, LDL, Cys C, and HCY in the high LDL/HDL group were higher than those in the low LDL/HDL group (P<0.001), while HDL was lower (P<0.001). In echocardiography, the left ventricular ejection fraction (LVEF) in the high LDL/HDL group was higher than that in the low LDL/HDL group (P=0.021). Compared with subjects in the medium LDL/HDL group, those in the high LDL/HDL group were younger (P=0.001) and had a higher BMI (P<0.001), and the proportion of diabetic patients was higher (P=0.008). In terms of laboratory tests, the blood glucose (P=0.003), Cys C (P=0.034), HCY (P=0.002), TG, TC, and LDL levels in the high LDL/HDL group were higher than those in the medium LDL/HDL group (P<0.001), while HDL levels were lower (P<0.001). For coronary angiography, the proportion of culprit vessels with TIMI blood flow grade 2 in the high LDL/HDL group was lower (P=0.013). As for pharmacotherapy, compared with the medium LDL/HDL group, statins (P=0.014) and spironolactone (P=0.040) were less frequently used in the high LDL/HDL group.

**Table 1:** Comparison of baseline data among the three groups.

	Low LDL/HDL (n=305)	Medium LDL/HDL (n=305)	High LDL/HDL (n=305)	P_1_	P_2_
Male (%)	255 (83.61)	273 (89.51)	267 (87.54)	0.205	0.526
Age (years)	60.14±10.86	58.60±11.13	55.51±10.98	<0.001	0.001
BMI (kg/m^2^)	23.36±2.60	23.93±2.59	24.72±2.92	<0.001	<0.001
HR (beats/min)	74.90±13.12	76.11±15.04	77.02±15.47	0.068	0.464
SBP (mmHg)	123.11±20.40	123.71±19.22	122.26±20.24	0.602	0.362
DBP (mmHg)	77.35±13.44	78.02±12.34	76.52±12.28	0.426	0.133
Smoking history (%)	195 (63.93)	222 (72.79)	218 (71.48)	0.057	0.787
Hypertension (%)	135 (44.26)	141 (46.23)	123 (40.33)	0.367	0.165
Diabetes mellitus (%)	43 (14.10)	32 (10.49)	56 (18.36)	0.187	0.008
Prior acute myocardial infarction (%)	15 (4.92)	19 (6.23)	19 (6.23)	0.597	1.000
Killip classification					
I (%)	178 (58.36)	203 (66.56)	212 (69.51)	0.005	0.487
II (%)	96 (31.48)	80 (26.23)	74 (24.26)	0.058	0.641
III (%)	15 (3.93)	16 (5.25)	11 (3.61)	0.548	0.432
IV (%)	16 (5.24)	6 (1.97)	8 (2.62)	0.143	0.788
Glucose (mg/dL)	136.15±63.08	134.86±57.24	152.22±84.20	0.008	0.003
Cr (mg/dL)	0.98±0.19	1.00±0.20	0.97±0.18	0.489	0.073
BUN (mg/dL)	14.49±5.32	15.00±5.26	14.59±5.50	0.808	0.351
TG (mg/dL)	112.18±49.75	141.14±69.09	181.12±105.29	<0.001	<0.001
TC (mg/dL)	132.78±28.61	155.96±41.04	185.95±65.72	<0.001	<0.001
LDL (mg/dL)	69.33±18.26	89.80±17.44	117.11±36.15	<0.001	<0.001
HDL (mg/dL)	45.35±11.63	39.53±7.25	35.20±8.35	<0.001	<0.001
Cys C (mg/dL)	0.10±0.04	0.13±0.07	0.15±0.11	<0.001	0.034
HCY (mg/dL)	0.33±0.16	0.37±0.13	0.44±0.15	<0.001	0.002
Hs-CRP (mg/dL)	0.99 (0.50-2.08)	0.78 (0.44-1.43)	0.82 (0.48-1.50)	0.464	0.415
Coronary angiography					
IRA					
LAD (%)	173 (56.72)	171 (56.07)	158 (51.80)	0.255	0.330
LCX (%)	30 (9.84)	26 (8.52)	28 (9.18)	0.890	0.887
RCA (%)	100 (32.79)	107 (35.08)	114 (37.38)	0.270	0.613
LM (%)	2 (0.66)	1 (0.33)	5 (1.64)	0.450	0.216
IRA TIMI flow grade					
0 (%)	127 (41.64)	139 (45.57)	144 (47.21)	0.192	0.745
1 (%)	10 (3.28)	8 (2.62)	7 (2.30)	0.624	1.000
2 (%)	17 (5.57)	28 (9.18)	12 (3.93)	0.447	0.013
3 (%)	151 (49.51)	130 (42.62)	142 (46.56)	0.517	0.370
Echocardiography					
LVEF (%)	52.22±11.94	53.97±10.92	54.39±11.24	0.021	0.635
LVEDD (mm)	53.23±5.96	53.03±6.15	53.01±6.25	0.662	0.969
LVESD (mm)	38.35±7.40	37.59±7.30	37.33±7.29	0.086	0.661
LVEDV (mL)	104.38±23.82	102.87±23.75	102.59±24.04	0.356	0.887
LVESV (mL)	56.21±21.18	53.47±20.24	52.92±20.32	0.051	0.735
Medications					
Aspirin (%)	304 (99.67)	296 (97.05)	300 (98.36)	0.216	0.418
Clopidogrel (%)	304 (99.67)	305 (100)	300 (98.36)	0.216	0.061
ACEI/ARB (%)	286 (93.77)	288 (94.43)	282 (92.46)	0.632	0.414
β-Blocker (%)	284 (93.11)	284 (93.11)	273 (89.51)	0.150	0.150
Statin (%)	291 (95.41)	295 (96.72)	280 (91.80)	0.097	0.014
Nitrate (%)	252 (82.62)	261 (85.57)	260 (85.25)	0.440	1.000
CCB (%)	277 (90.82)	270 (88.52)	273 (89.51)	0.684	0.796
Spironolactone (%)	263 (86.23)	279 (91.48)	262 (85.90)	1.000	0.040
Diuretic (%)	290 (95.08)	296 (97.05)	286 (93.77)	0.597	0.080
Digoxin (%)	300 (98.36)	302 (99.02)	300 (98.36)	1.000	0.725

Data are reported as the means±SD, median (IQR), or n (%). Chi-squared test, independent-sample*t*-test, and Mann-Whitney U test were used for statistical analysis. P_1_: high LDL/HDL group compared with low LDL/HDL group*;* P_2_: high LDL/HDL group compared with medium LDL/HDL group; BMI: body mass index; HR: heart rate; SBP: systolic blood pressure; DBP: diastolic blood pressure; Cr: creatinine; BUN: blood urea nitrogen; TG: triglycerides; TC: total cholesterol; LDL: low-density lipoprotein; HDL: high-density lipoprotein; Cys C: cystatin C; HCY: homocysteine; Hs-CRP: highly sensitive C-reactive protein; IRA: infarct-related artery; LAD: left anterior descending artery; LCX: left circumflex; RCA: right coronary artery; LM: left main; LVEF: left ventricular ejection fraction; LVEDD: left ventricular end-diastolic dimension; LVESD: left ventricular end-systolic dimension; LVEDV: left ventricular end-diastolic volume; LVESV: left ventricular end-systolic volume; ACEI: angiotensin-converting enzyme inhibitor; ARB: angiotensin receptor blocker; CCB: calcium channel blocker.

### Clinical endpoints

The comparison of clinical endpoints among the three groups is shown inTable 2. There were no differences in all-cause death, MACEs, cardiac death, nonfatal myocardial infarction, heart failure, or stroke between the high LDL/HDL group and either the low LDL/HDL group or the medium LDL/HDL group (P>0.05). The rate of coronary revascularization in the high LDL/HDL group was significantly higher than that in the low LDL/HDL (P=0.001) and medium LDL/HDL groups (P=0.010).

**Table 2:** Comparison of all-cause death, MACEs, and coronary revascularization among the three groups.

	Low LDL/HDL (n=305)	Medium LDL/HDL (n=305)	High LDL/HDL (n=305)	P_1_	P_2_
All-cause death (%)	63 (20.66)	50 (16.39)	48 (15.74)	0.115	0.825
MACEs (%)	142 (46.56)	141 (46.23)	157 (51.48)	0.257	0.224
Cardiac death (%)	50 (16.39)	38 (12.46)	38 (12.46)	0.167	1.000
Coronary revascularization (%)	53 (17.38)	59 (19.34)	87 (28.52)	0.001	0.010
Nonfatal acute myocardial infarction (%)	11 (3.61)	16 (5.25)	13 (4.26)	0.677	0.568
Heart failure (%)	21 (6.89)	24 (7.87)	21 (6.89)	1.000	0.642
Stroke (%)	10 (3.28)	13 (4.26)	12 (3.93)	0.664	0.838

Data are reported as the n (%). Chi-squared test was used for statistical analysis. P_1_: high LDL/HDL group compared with low LDL/HDL group; P_2_: high LDL/HDL group compared with medium LDL/HDL group; LDL: low-density lipoprotein; HDL: high-density lipoprotein; MACEs: major adverse cardiovascular events.

### Kaplan-Meier cumulative hazard curves

**Figure 1:**
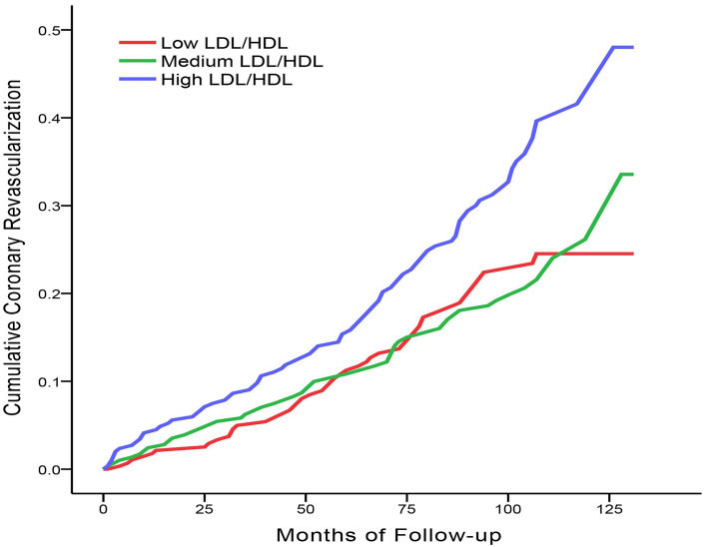
Kaplan-Meier cumulative hazard curves of coronary revascularization in the three groups. Log-rank test showed that the chi-squared value between the low LDL/HDL group and the high LDL/HDL group was 7.290, P=0.007, and the chi-squared value between the medium LDL/HDL group and the high LDL/HDL group was 8.105, P=0.004. LDL: low-density lipoprotein; HDL: high-density lipoprotein.

K-M cumulative hazard curves of clinical endpoints in the three groups are shown inFigure 1 and Supplementary Figure S1. The hazards of all-cause death, MACEs, cardiac death, nonfatal acute myocardial infarction, heart failure, and stroke in the high LDL/HDL group were not significantly different from those in the low LDL/HDL or medium LDL/HDL groups (P>0.05) (Supplementary Figure S1). However, the hazard of coronary revascularization in the high LDL/HDL group was significantly higher than that in the low LDL/HDL (P=0.007) and medium LDL/HDL groups (P=0.004) (Figure 1).

### Multivariate Cox regression analysis

**Figure 2:**
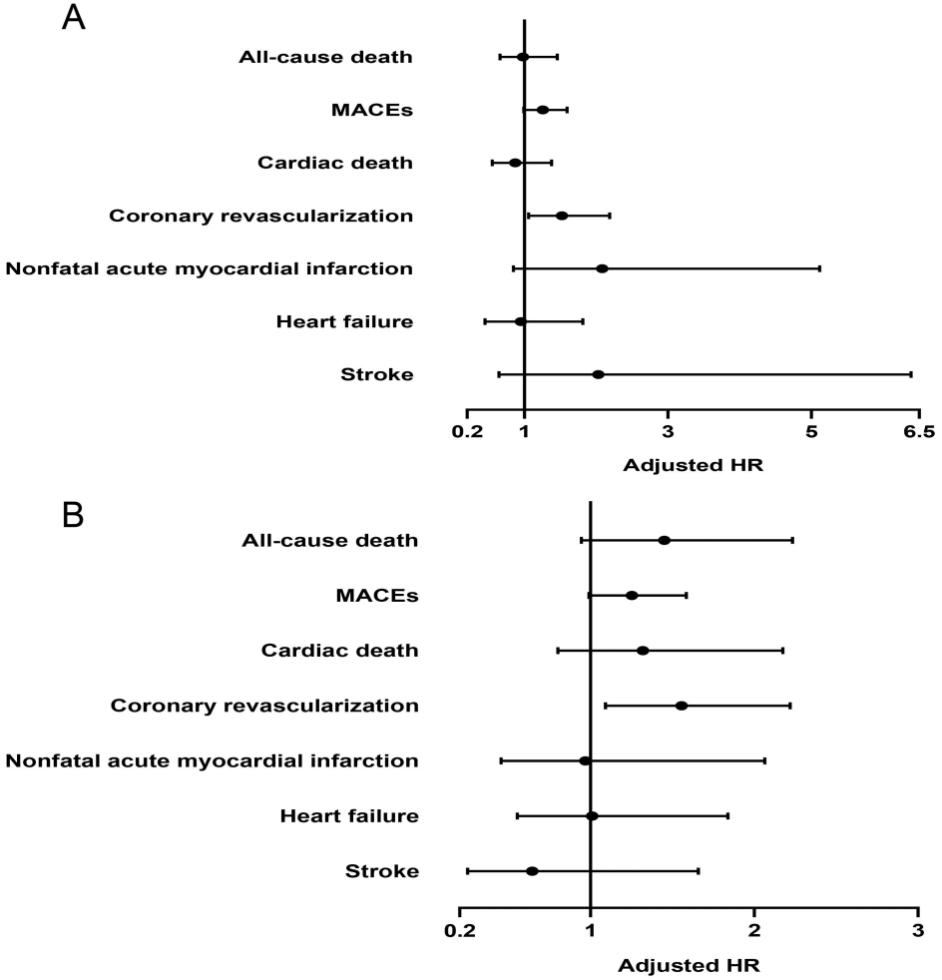
Forest plots for multivariate Cox regression analysis of the LDL/HDL ratio and clinical endpoints. **A**, High LDL/HDL compared with low LDL/HDL; **B**, High LDL/HDL compared with medium LDL/HDL. LDL: low-density lipoprotein; HDL: high-density lipoprotein; MACEs: major adverse cardiovascular events.

The Cox regression analysis of the LDL/HDL ratio and clinical endpoints in STEMI patients is shown inTable 3,Figure 2, and Supplementary Table S1. Univariate Cox regression analysis showed that high LDL/HDL compared to low LDL/HDL (HR=1.574, 95%CI: 1.115-2.222, P=0.010) and medium LDL/HDL (HR=1.634, 95%CI: 1.158-2.307, P=0.005) was associated with higher risk of coronary revascularization. Furthermore, there was a linear relationship between the LDL/HDL ratio and coronary revascularization incidence. With every unit increase in the LDL/HDL ratio, the HR of coronary revascularization was 1.267 (95%CI: 1.060-1.514, P=0.009) (Supplementary Table S1). After adjusting for sex, age, hypertension, diabetes, smoking history, prior acute myocardial infarction, systolic blood pressure, diastolic blood pressure, and statin use, high LDL/HDL compared to low LDL/HDL (HR=1.520, 95%CI: 1.057-2.185, P=0.024) and medium LDL/HDL (HR=1.556, 95%CI: 1.091-2.220, P=0.015) was an independent risk factor for long-term coronary revascularization in STEMI patients who underwent PCI. Furthermore, the association of the LDL/HDL ratio, as a continuous variable, with coronary revascularization remained significant, with an adjusted HR of 1.231 (95%CI: 1.023-1.482) per unit increase in the LDL/HDL ratio (P=0.028) (Table 3 andFigure 2).

**Table 3:** Multivariate Cox regression analysis of the LDL/HDL ratio and clinical endpoints in STEMI patients.

	High LDL/HDL^a^			High LDL/HDL^b^			Overall tendency		
	HR	95%CI	P	HR	95%CI	P	HR	95%CI	P
All-cause death	0.980	0.659-1.457	0.920	1.451	0.944-2.233	0.090	0.983	0.800-1.208	0.871
MACEs	1.256	0.989-1.595	0.062	1.253	0.991-1.585	0.059	1.126	0.999-1.270	0.053
Cardiac death	0.870	0.549-1.378	0.554	1.319	0.800-2.175	0.278	0.917	0.725-1.160	0.472
Coronary revascularization	1.520	1.057-2.185	0.024	1.556	1.091-2.220	0.015	1.231	1.023-1.482	0.028
Nonfatal acute myocardial infarction	2.081	0.847-5.112	0.110	0.968	0.454-2.064	0.932	1.392	0.923-2.101	0.115
Heart failure	0.951	0.449-1.814	0.879	1.009	0.553-1.839	0.978	1.044	0.766-1.422	0.785
Stroke	2.028	0.644-6.386	0.227	0.643	0.249-1.659	0.361	1.189	0.723-1.957	0.495

Adjustments were applied for significant influential baseline characteristics of sex, age, hypertension, diabetes, smoking history, prior acute myocardial infarction, systolic blood pressure, diastolic blood pressure, and statin use. Cox regression analysis was used for statistical analysis. ^a^high LDL/HDL compared with low LDL/HDL; ^b^high LDL/HDL compared with medium LDL/HDL; HR: hazard ratio; LDL: low-density lipoprotein; HDL: high-density lipoprotein; MACEs: major adverse cardiovascular events.

### Correlations between the LDL/HDL ratio and oxidation biomarkers

There were weak positive correlations between the LDL/HDL ratio and both Cys C (r=0.354, P<0.001) and HCY (r=0.336, P=0.001).

## Discussion

To our knowledge, this is the first report that the LDL/HDL ratio can predict the long-term risk of coronary revascularization in STEMI patients undergoing PCI. A high LDL/HDL ratio was an independent risk factor for coronary revascularization in STEMI patients, and the risk of coronary revascularization significantly increased when LDL/HDL≥2.595.

Previous studies have shown that the LDL/HDL ratio can predict the risk of adverse cardiovascular events. After 23 years of follow-up of 2616 healthy subjects, the Kuopio Ischemic Heart Disease Risk Factor (KIHD) study found that neither LDL nor HDL had a correlation with the occurrence of sudden cardiac death (SCD) but that the LDL/HDL ratio was an independent risk factor for SCD. The risk of SCD in patients with LDL/HDL>4.22 was approximately two-fold that in patients with LDL/HDL≤2.3, suggesting that the LDL/HDL ratio is better than LDL or HDL alone for predicting the risk of SCD (12). A case-control study showed that the LDL/HDL ratio was an independent risk factor for acute myocardial infarction in male patients. An LDL/HDL ratio higher than 3.36 was associated with a significantly greater risk of acute myocardial infarction (7). Similarly, another cohort study found that increased LDL/HDL was an independent risk factor for adverse cardiovascular events in patients who underwent PCI and drug-eluting stent implantation (8). However, there is no report on whether the LDL/HDL ratio can be a predictor of long-term cardiovascular adverse events in STEMI patients. Our experiment found through a cohort study that the LDL/HDL ratio can predict the long-term risk of coronary revascularization in STEMI patients undergoing PCI. When LDL/HDL was ≥2.595, the risk of coronary revascularization in STEMI patients increased significantly. After adjusting for factors such as sex, age, and medical history, a high LDL/HDL ratio was an independent risk factor for long-term coronary revascularization in STEMI patients. This study also found that there was no correlation between the LDL/HDL ratio and the risk of MACEs. We believe that this result may have been due to the fact that the LDL/HDL ratio was not significantly related to the risks of other clinical endpoints included in MACEs, including cardiac death, nonfatal acute myocardial infarction, heart failure, and stroke, thereby weakening the relationship between the LDL/HDL ratio and MACEs.

The exact mechanism between the LDL/HDL ratio and long-term coronary revascularization in STEMI patients has not been elucidated. Previous studies have confirmed that the occurrence of adverse cardiovascular events, including coronary revascularization, is related to biomarkers such as myeloperoxidase (MPO), glutathione peroxidase, and gamma-glutamyl transferase (GGT), which can reflect the level of human oxidation reaction (13-15), and the LDL/HDL ratio can also reflect the level of oxidation reaction (6). Therefore, we speculate that the LDL/HDL ratio can reflect the level of oxidation reactions to predict the long-term risk of coronary revascularization in STEMI patients.

With in-depth research on coronary heart disease, a large volume of evidence has shown that oxidation reactions play an important role in the occurrence and progression of coronary heart disease. Reactive oxygen species (ROS), including superoxide anions (O_2_^-^) and hydroxyl radicals (-OH), etc., are oxygen single-electron reduction products produced in enzymatic reactions catalyzed by enzymes such as cyclooxygenase and peroxidase (16). LDL generates oxidized low-density lipoprotein (ox-LDL) through the action of ROS, and ox-LDL can form foam cells after being engulfed by macrophages (5). In addition, ROS can activate matrix metalloproteinases (MMPS) and cause atherosclerotic plaque rupture (17), advancing coronary heart disease into acute coronary syndrome. Therefore, biomarkers that can reflect the level of oxidation reaction should be related to the long-term prognosis of STEMI patients. At the same time, LDL oxidized by ROS and phagocytosed by macrophages can produce ROS, which in turn enhances the oxidation reaction (18), and HDL can reduce ROS by inhibiting the activation of generated endothelial nitric oxide synthase (eNOS), reducing the level of oxidation reaction (19). Studies have confirmed that the LDL/HDL ratio increases by 1 and the ox-LDL/HDL ratio, which reflects the oxidation reaction of the human body, increases by 0.831. When the LDL/HDL ratio decreases by 1.014, the superoxide dismutase (SOD), which represents antioxidant levels of the human body, will increase by 1 U/mL; when the LDL/HDL ratio decreases by 0.023, the glutathione peroxidase (GPx3), which also represents antioxidant levels of the human body, will increase by 1 nmol/min/mL (6). Therefore, the LDL/HDL ratio can represent the level of human oxidation.

Cys C is produced by the nucleus and is released into the blood, which can be freely filtered from the glomerulus. Studies have shown that the level of Cys C is positively correlated with the infarct size of patients with acute myocardial infarction (20). Meanwhile, Cys C, the oxidation site of peroxide, can increase the level of the oxidation reaction (21). As a sulfur-containing amino acid, HCY is an important intermediate product in the metabolism of methionine and cysteine. Studies have shown that HCY can enhance the oxidation reaction through endoplasmic reticulum stress and the Nox4/NF-κB pathway (22). There is no report about the correlation between the LDL/HDL ratio and Cys C or HCY. Our results showed that the LDL/HDL ratio was positively correlated with both Cys C and HCY, which provides further support for the view that the LDL/HDL ratio can reflect the level of oxidation. In summary, the LDL/HDL ratio may reflect the level of human oxidation to predict the long-term risk of coronary revascularization in STEMI patients, but this mechanism needs confirmation with further research.

In spite of the interesting findings of the study, several shortcomings should be noted. First, the study is limited in its retrospective and observational nature. Second, the results may be confounded by familial hypercholesterolemia and other unknown or unmeasured factors. Third, the ratio of loss to follow-up in this study was relatively high, which may have introduced loss-to-follow-up bias. Finally, the last follow-up was performed via telephone and medical records, which may have also introduced bias. In our next work, we will establish a larger cohort and conduct more rigorous management to verify the prognostic value of the LDL/HDL ratio for the long-term risk of cardiovascular adverse events in STEMI patients undergoing PCI.

In conclusion, our study suggests that the LDL/HDL ratio can predict the long-term risk of coronary revascularization in STEMI patients undergoing PCI. A high LDL/HDL ratio was an independent risk factor for coronary revascularization in STEMI patients. The risk of coronary revascularization in patients with LDL/HDL ≥2.595 was significantly increased.

## Supplementary Material

Click to view [pdf].
